# The Day Camp for Tuberculosis Patients

**Published:** 1911-07

**Authors:** 


### The Day Camp for Tuberculosis Patients

WITH the opening of the Day Camp for tubercular patients at Main Street and LaSalle avenue, Buffalo, during the month, the necessity for the raising of funds to carry on this excellent work of relief becomes apparent. Elsewhere is published a statement of the resources of the camp and its financial necessities. Money is needed urgently to carry on relief for this most deserving class of patients and the results achieved in past years more than justify the public for the money to continue. The capacity of the camp is much too small. There are applications on hand numbering several times the capacity of the association's outfit and money is needed. The county officials show a definite disinclination to better the condition of the tubercular-ridden, and until they do, the care, the comfort and the control rests with individual charity.

